# Association between ranitidine use with potential NDMA impurities and risk of cancer in Korea

**DOI:** 10.1038/s41598-022-26691-0

**Published:** 2022-12-27

**Authors:** Kyung-In Joung, Jung Eun Hwang, In-Sun Oh, Sung-il Cho, Ju-Young Shin

**Affiliations:** 1 School of AI Healthcare, College of Integrated Health Sciences, Cha University, Pocheon, Republic of Korea; 2 School of Pharmacy, Sungkyunkwan University, Suwon, Gyeonggi-Do Republic of Korea; 3 Department of Biohealth Regulatory Science, Sungkyunkwan University, Suwon, Gyeonggi-Do Republic of Korea; 4 Department of Epidemiology, Biostatistics, and Occupational Health, McGill University, Montreal, QC Canada; 5 Centre for Clinical Epidemiology, Lady Davis Research Institute - Jewish General Hospital, 3755 Cote Ste-Catherine, H-410.1, Montreal, QC Canada; 6 Department of Public Health Science, Graduate School of Public Health and Institute of Health and Environment, Seoul National University, Seoul, Republic of Korea; 7 (16419) Department of Clinical Research Design & Evaluation, Samsung Advanced Institute for Health Sciences & Technology, Sungkyunkwan University, Seoul, Republic of Korea

**Keywords:** Cancer epidemiology, Cancer, Risk factors

## Abstract

N-Nitrosodimethylamine (NDMA) detected above the acceptable level in ranitidine products has been a great global concern. To examine the risk of cancer among people treated with ranitidine, we conducted a cohort study using the National Health Insurance Service-National Sample Cohort data (2002–2015) of South Korea. Patients were aged 40 or above as of January 2004 and began receiving ranitidine or other histamine-2 receptor antagonist (H2RA), active comparator, without a history of H2RAs prescription during the prior 2-years. The lag time was designated up to 6 years. The outcomes were an overall incident cancer risk and the risk of major single cancers during the follow-up. The association between ranitidine use and cancer risk was examined by Cox regression model. After exclusion and propensity score matching, 25,360 patients were available for analysis. The use of ranitidine was not associated with the overall cancer risk and major individual cancers [overall cancer: incidence rate per 1000 person-years, 2.9 vs 3.0 among the ranitidine users and other H2RAs users, respectively; adjusted hazard ratio (HR) and 95% confidence interval (95% CI) for all cancers, 0.98 (0.81–1.20)]. The higher cumulative exposure to ranitidine did not increase the cancer risk. Given the insufficient follow-up period, these findings should be interpreted carefully.

## Introduction

N-Nitrosodimethylamine (NDMA) is a volatile chemical belonging to the nitrosamine class of compounds. It is a by-product of manufacturing processes involving alkylamines that leaches into the air, water, and soil. Human exposure to NDMA may occur through tobacco smoke, food items, especially nitrite-preserved foods, such as cured meats, and various household goods^1,2^. NDMA can also form in the stomach endogenously during digestion of alkylamine-containing foods^3^. It is well-established that NDMA is carcinogenic in animals^2,4–6^. Although data in humans is scarce, based on the laboratory studies, NDMA has been classified as “possibly carcinogenic to humans (group 2A)” by the International Agency for Research on Cancer (IARC)^7^.

The most significant issue in recent years related to NDMA seems to be pharmaceutical contamination. In 2018, NDMA was detected above the acceptable level in pharmaceutical products containing valsartan, an antihypertensive drug^8^. It was subsequently detected in products containing ranitidine^9^, nizatidine^10^, and metformin^11^ in 2019. In particular, NDMA impurities in ranitidine, a histamine-2 receptor antagonist (H2RA) used to treat and prevent gastric ulcer has raised great concern, considering ranitidine is widely used both as an over-the-counter and prescription drug. Besides, a recent laboratory study using liquid chromatography-high resolution mass spectrometry suggested that ranitidine may be a significant source of NDMA under simulated gastric conditions^12^. Some evidence suggests that NDMA can arise from the degradation of ranitidine itself with increasing levels over its shelf life. Ranitidine is also suspected of producing NDMA in the human body. These considerations necessitate a study of where ranitidine use itself is linked to cancer risk, regardless of whether NDMA was detected in individual ranitidine products^13,14^.

In South Korea, all seven ranitidine-based raw ingredients were inspected promptly after the U.S. Food and Drug Administration (FDA) announcement concerning the NDMA impurities in ranitidine drug substances. All of them were found to exceed the domestic acceptable daily limit (0.16 ppm), while the variation is considerable from undetected to 53 ppm depending on the test sample. Accordingly, the Ministry of Food and Drug Safety (MFDS) prohibited the manufacturing, marketing, and prescription of 269 finished products. At that time, the number of patients taking ranitidine reached 1.44 million^15^. Contamination of NDMA in ranitidine is a global issue. The U.S. FDA has determined that the impurities in NDMA in ranitidine products increases over time to unacceptable levels and, as the latest step, has requested manufacturers withdraw all ranitidine drugs from the market immediately. Similarly, in September 2020, EMA suspended all ranitidine medicines in the EU to the presence of low levels of an impurity of NDMA^13^.

Studies examined overall cancer risk among ranitidine users in terms of potential NDMA impurities are scarce^16–19^. While these studies found no association between ranitidine and risk of cancer, they have limitations such as a short follow-up and generalization^16^, insufficient control of potential confounding variables^17^, and use of self-reported exposure^18^. Studies on the association between exposure to ranitidine and specific cancer site were mostly focused on gastric cancer^19–21^, and the relationship between ranitidine and risk of other single cancer were less investigated^22,23^. While no evidence of increased risk of gastric cancer was provided by existing studies^19–22^, results regarding the risk of bladder cancer are conflicting^22,23^.

Korea is recognized for highly prevalent prescriptions for acid-suppressing drugs including ranitidine and a wide variety range of ranitidine products demanding a more thorough investigation^24^. Besides, Korea has the highest rate of gastric cancer in the world^25^. The present study aimed to estimate the risk of overall cancer and nine cancers by specific sites among people treated with ranitidine with NDMA impurities compared with those treated with other H2RAs.

## Materials and methods

### Data source

The National Health Insurance Service-National Sample Cohort (NHIS-NSC), a population-based cohort established by the NHIS in South Korea, was used in this study^26^. This cohort included detailed information regarding medical utilization of about 1 million people as of 2006 (corresponding to about 2.0% of the total eligible Korean population), who were followed for 14 years starting in 2002 until 2015, unless participants’ eligibility was disqualified due to death or emigration. The National Health Insurance (NHI) is a single-insurer system with complete universal healthcare coverage in Korea since 2000^26^. The medical-treatment database includes details of electronic medical-treatment bills, diagnoses, and prescriptions. Information on the database can be obtained from the NHI Sharing Service website (https://nhiss.nhis.or.kr/bd/ab/bdaba005iv.do). Information regarding all medical products licensed and distributed in Korea was obtained from the Health Insurance Review and Assessment Service (HIRA), a government-affiliated organization that reviews and evaluates healthcare costs and healthcare service quality (https://www.hira.or.kr/rd/insuadtcrtr/InsuAdtCrtrList.do?pgmid=HIRAA030069000400).

### Study design and population

**Figure 1:**
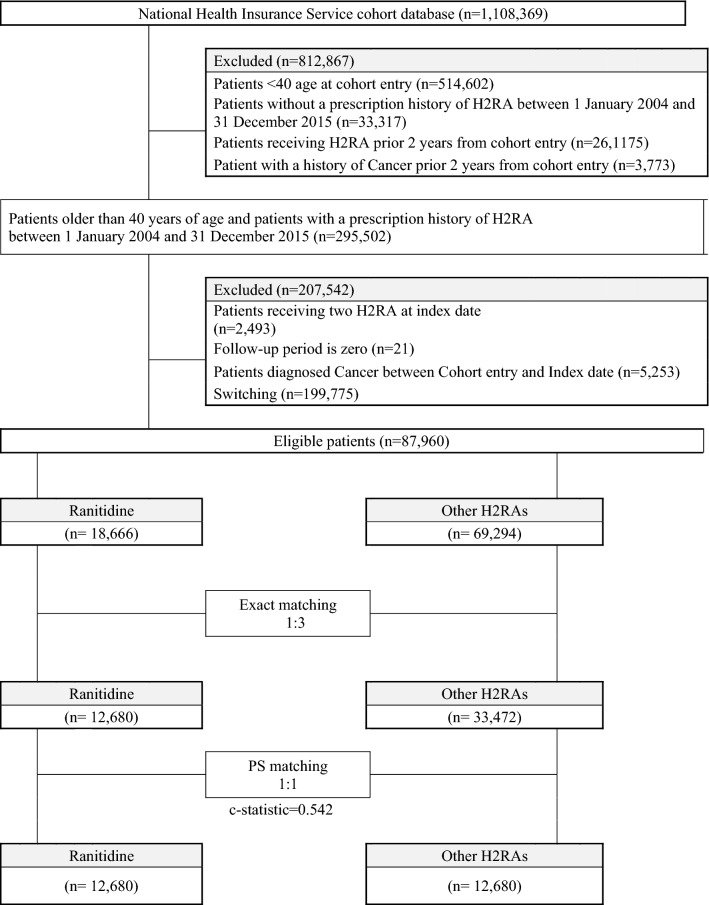
Selection of study participants from the National Health Insurance Service-National Sample Cohort. *H2RA* histamine-2 receptor antagonist.

This was a retrospective cohort study that utilized the NHIS-NSC data (2002–2015). We selected those who were over 40 years of age as of January 1, 2004 (the cohort entry date) as the study subjects to increase the power of individual cancer analysis by securing cases, and to consider the difference in the etiology of cancer in children and adults. New users of ranitidine or other H2RAs were defined as those who took these drugs for the first time between January 1, 2004 and December 31, 2015, without a history of H2RAs prescription two years prior (2002–2003). Patients who had been diagnosed with any cancer during the preceding two-year period were excluded. The exclusion of two years was not a sufficient period to screen only new cancer patients. However, it was determined not to further reduce the period for following up cancer occurrence, considering NHIS-NSC data was available only from 2002 to 2015. Patients who had once switched between ranitidine and an active control drug were also excluded. In addition, patients who received more than one H2RA on the index date, and whose index date was the last day of the study, were excluded. Patients diagnosed with cancer between cohort entry and index date were also excluded. Figure 1 illustrates the algorithm for the selection of study participants.

### Exposure definition

While our primary exposure of interest was the use of NDMA-containing ranitidine, we used ranitidine prescription as an alternative measure for the following considerations: First, the MFDS investigated all of the seven active pharmaceutical ingredients(APIs) manufacturers in circulation, and as NDMA was detected in all of them, it was acknowledged that all finished ranitidine products circulating in Korea had potential NDMA impurities and MFDS suspended all ranitidine medicines^15^. Second, it has been suggested that ranitidine is easily decomposed during storage to form NDMA due to its inherently unstable nature and tertiary amine structure^14^. Finally, no approach for determining exposure to NDMA-contaminated ranitidine currently. An active comparator was defined as a new user of any of the following H2RA other than ranitidine: cimetidine, lafutidine, nizatidine, famotidine, and roxatidine. Although NDMA was detected in nizatidine, we did not exclude nizatidine users in the control group since the level only slightly exceeded the authorities' criterion of 0.32 ppm (range: 0.34 ppm ~ 1.43 ppm), which corresponds to 1/37 of the detection amount in ranitidine based on the maximum detection amount^27^. The index date was designated as the first prescription date of ranitidine or other H2RAs.

The cumulative duration of ranitidine use was calculated by summing up all the prescription days, regardless of continuity (< 14 days, 15–30 days, 31–60 days, 61–90 days, 91–180 days, and > 180 days). The cumulative dose of ranitidine in defined daily dose (DDD) provided by World Health Organization (WHO) was also measured, and subjects were categorized into three groups (< 6 DDD, 6–50 DDD, and > 50 DDD).

### Outcome and follow-up

The primary outcome was a composite of all cancers during the follow-up period, identified by the International Classification of Disease 10^th^ Revision (ICD-10) (C00-C97). The cancer outcome was double-checked using the V code, a South Korea-specific classification to validate cancer for reimbursement policy purposes. Subjects were followed up from the beginning to diagnosis of cancer, death, loss to follow-up, or December 31, 2015, whichever occurred first. The risk of individual cancers for which animal studies or observational studies previously explored was also assessed. To reflect the induction and latent period until cancer was diagnosed and exclude the possibility of protopathic bias (reverse causation), the lag time was set to two years, and cancer that was diagnosed within the lag time was censored.

### Potential confounders

The following potential confounding variables were included as covariates: basic demographic variables, such as age, sex, income level, region, insurance type; Charlson comorbidity index; polypharmacy (number of average daily prescribed drugs ≥ 5); index year; medical histories, such as chronic obstructive pulmonary disease, obesity, alcohol-related disease, hypertension, severe liver disease, diabetes mellitus, kidney disease, disorders of the gallbladder, biliary tract, and pancreas; congestive heart failure; ischemic heart disease; atrial fibrillation; stroke; ulcerative colitis; and co-medications, such as 5-alpha-reductase inhibitors, glucocorticoids for systemic use, hormone replacement therapy, low-dose aspirin, non-aspirin non-steroidal anti-inflammatory drugs, spironolactone, statins, angiotensin-2-antagonists, antidepressants, antipsychotics, and proton pump inhibitors. Comorbidity and co-medication were defined according to previous diagnoses and the prescription of drugs within one year before the index date. The ICD-10 codes used to define the comorbidities are presented in the Supplementary Table 1.

### Propensity score matching

After exact 1:3 matching of sex, age, and duration from cohort entry to index date, the propensity scores were estimated for receiving ranitidine prescription by multiple logistic regressions for the all of the aforementioned potential confounding variables (all variables presented in the Table 1). Model discrimination was assessed using C-statistic^28^. Matching was performed using the Greedy 8 → 1 digit match macro with the estimated propensity score^29^. The standardized difference was used to compare baseline characteristics of patients treated with ranitidine and other H2RAs, and defined imbalance as an absolute value greater than 0.1^30^.

**Table 1:** Baseline characteristics of ranitidine users versus other H2RAs users in overall cohort and propensity matched cohort.

Characteristic	Overall cohort			Propensity-based matched cohort		
	Ranitidine n = 18 666 (%)	Other H2RA n = 69 294 (%)	Standardized difference	Ranitidine n = 12 680 (%)	Other H2RA n = 12 680 (%)	Standardized difference
Sex. males	12 112 (64.9)	41 838 (60.4)	0.093	8199 (64.7)	8201 (64.7)	0.000
Duration of follow up (years, median [IQR])	4.16 [2.02–6.83]	8.47 [5.33–10.28]		5.49 [3.02–7.91]	5.79 [3.32–8.16]	
Age at index (mean ± SD)	56.2 ± 12.9	55.4 ± 13.0	0.062	55.9 ± 12.8	55.7 ± 12.7	0.021
**Type of health insurance**			0.044			0.007
Health insurance	17 914 (96.0)	67 070 (96.8)		12 155 (95.9)	12 136 (95.7)	
Medical aid	752 (4.0)	2224 (3.2)		525 (4.1)	544 (4.3)	
**Income level**			0.016			0.015
1st quartile (most deprived)	2998 (16.1)	10 847 (15.7)		1992 (15.7)	2018 (15.9)	
2nd quartile	4882 (26.2)	17 941 (25.9)		3333 (26.3)	3257 (25.7)	
3rd quartile	6467 (34.6)	24 481 (35.3)		4472 (35.3)	4524 (35.7)	
4th quartile (most affluent)	4319 (23.1)	16 025 (23.1)		2883 (22.7)	2881 (22.7)	
**Charlson comorbidity index**			0.083			0.038
0	17 354 (93.0)	62 974 (90.9)		11 728 (92.5)	11 851 (93.5)	
1	768 (4.1)	4054 (5.9)		536 (4.2)	464 (3.7)	
2 +	544 (2.9)	2266 (3.3)		416 (3.3)	365 (2.9)	
**Region**			0.072			0.007
Capital area	8191 (43.9)	32 881 (47.5)		5504 (43.4)	5457 (43.0)	
Other regions	10 475 (56.1)	36 413 (52.5)		7176 (56.6)	7223 (57.0)	
**Polypharmacy**			0.007			0.010
< 5 medications	18 643 (99.9)	69 191 (99.9)		12 666 (99.9)	12 670 (99.9)	
≥ 5 medications	23 (0.1)	103 (0.1)		14 (0.1)	10 (0.1)	
**Comorbidities**						
Diseases of the digestive system	1526 (8.2)	9068 (13.1)	0.160	1160 (9.1)	1099 (8.7)	0.017
Hypertension	1268 (6.8)	5071 (7.3)	0.021	873 (6.9)	718 (5.7)	0.050
Diabetes	631 (3.4)	2 533 (3.7)	0.015	431 (3.4)	352 (2.8)	0.036
Ischaemic heart disease	230 (1.2)	835 (1.2)	0.002	163 (1.3)	136 (1.1)	0.020
Congestive heart failure	122 (0.7)	340 (0.5)	0.022	93 (0.7)	100 (0.8)	0.006
Disorders of gall bladder, biliary tract, and pancreas	33 (0.2)	178 (0.3)	0.017	23 (0.2)	24 (0.2)	0.002
Kidney diseases	118 (0.6)	506 (0.7)	0.012	89 (0.7)	81 (0.6)	0.008
Atrial fibrillation	43 (0.2)	102 (0.1)	0.019	31 (0.2)	31 (0.2)	0.000
Severe liver disease	224 (1.2)	999 (1.4)	0.021	172 (1.4)	151 (1.2)	0.015
Alcohol-related disease	76 (0.4)	427 (0.6)	0.029	58 (0.5)	47 (0.4)	0.014
Stroke	11 (0.1)	19 (0.0)	0.015	7 (0.1)	6 (0.0)	0.003
Ulcerative colitis	12 (0.1)	53 (0.1)	0.005	9 (0.1)	11 (0.1)	0.006
Chronic pulmonary disease	560 (3.0)	3351 (4.8)	0.095	392 (3.1)	339 (2.7)	0.025
Obesity	7 (0.0)	38 (0.1)	0.008	5 (0.0)	6 (0.0)	0.004
Chronic liver disease	46 (0.2)	218 (0.3)	0.013	37 (0.3)	26 (0.2)	0.017
**Co-medications**						
Angiotensin-II antagonists	388 (2.1)	1402 (2.0)	0.004	259 (2.0)	207 (1.6)	0.031
Proton pump inhibitor	44 (0.2)	154 (0.2)	0.003	38 (0.3)	40 (0.3)	0.003
Statins	262 (1.4)	1035 (1.5)	0.008	178 (1.4)	132 (1.0)	0.033
Low-dose aspirin	316 (1.7)	1345 (1.9)	0.019	211 (1.7)	166 (1.3)	0.029
5α-reductase inhibitors	39 (0.2)	168 (0.2)	0.007	29 (0.2)	26 (0.2)	0.005
Glucocorticoids for systemic use	1 617 (8.7)	9835 (14.2)	0.174	1159 (9.1)	1102 (8.7)	0.016
Non-aspirin NSAIDs	4205 (22.5)	22 200 (32.0)	0.215	3009 (23.7)	2941 (23.2)	0.013
Hormone replacement therapy	64 (0.3)	286 (0.4)	0.011	45 (0.4)	37 (0.3)	0.011

*H2RA* histamine-2 receptor antagonist, *IQR* interquartile range, *NSAIDs* non-steroidal anti-inflammatory drugs.

### Stratified analysis and sensitivity analysis

Stratified analyses were conducted according to sociodemographic factors (sex, age, insurance type, income level, and region), comorbidities such as hypertension, diabetes, severe liver disease, chronic pulmonary disease, and comedications, including glucocorticoids for systemic use and non-aspirin non-steroidal anti-inflammatory drugs (NSAIDs). In the stratified design, interactions between ranitidine use and each stratification variable were calculated based on the additive model and presented as a *p*-value. Sensitivity analyses were performed to examine the robustness of the primary result and to avoid any biases in causality. First, different lag-times were applied, such as no-lag, two years (primary analysis), four years, and six years. Second, the type of intention-to-treat observational study was analyzed to closely emulate a randomized controlled trial^31,32^, wherein subjects who switched between therapies were not excluded. Third, the person-time of patients diagnosed with cancer during the two-year lag-time period was excluded from the person-time summation to prevent possible underestimation of cancer risk. Fourth, to limit the inclusion of non-compliant individuals, the analysis was performed only for patients who received at least two prescriptions. Finally, since several studies have demonstrated that increasing the look-back period improves the precision in identifying comorbid diseases^33^, we extended the look-back period for comorbidities and co-medications to 2 years.

### Supplementary analysis

We calculated attributable risk (AR) and population attributable risk (PAR) to quantify both the excess risk due to the exposure and the proportion of all incident cancers in the population that could be attributed to the exposure.

### Statistical analysis

Descriptive statistics were used to summarize the characteristics of ranitidine users and other H2RAs users at cohort entry^34^. The crude incidence rates per 1000 person-years with 95% confidence intervals (95% CIs) were calculated based on the Poisson distribution, overall and for each exposure category. Cox proportional hazards models were used to estimate adjusted hazard ratios (HRs) and 95% CIs for cancer associated with ranitidine use compared with other H2RAs use. The Schoenfeld residuals were examined to test proportional hazard assumption^35^. All data were analyzed using the SAS statistical application program (Version 9.4, SAS Institute Inc, NC, USA).

### Ethical approval

This study was approved by the institutional review board of Sungkyunkwan University (No SKKU 2019-12-009), which waived the requirement for informed consent as only deidentified data were used in this study.

## Results

In the overall cohort, 18,666 ranitidine users and 69,294 other H2RA (other than ranitidine) users met the study criteria. After propensity score estimation and one-to-one matching, the cohort included 12,680 ranitidine users and 12,680 other H2RA users (c-statistic: 0.543). The median follow-up period was 5.49 years and 5.79 years in ranitidine users and other H2RA users, respectively. Table 1 provides the baseline characteristics of ranitidine users and H2RA users in the overall cohort and propensity score-matched cohort. All the standardized difference scores in the propensity based matched cohort were less than 0.1 as an absolute value. Figure 1 illustrates the algorithm for the selection of study participants. Table 2 presents the risk for all cancers and individual cancer associated with the use of ranitidine. The use of ranitidine was not associated with all cancers or any type of cancer when compared with H2RAs use. Incidence rate per 1000 person-years was 2.9 and 3.0 among the ranitidine users and other H2RAs users, respectively. The adjusted HR and 95% CI for all cancers were 0.95 (0.83–1.09) in the overall cohort and 0.98 (0.81–1.20) in the propensity score-matched cohort. In both the overall and propensity score-matched cohorts, the risk of kidney cancer related to ranitidine use appeared to be greater than with other H2RAs use, but there was no statistical significance [HR (95% CI), 1.49 (0.61–3.61) in the overall cohort; 2.65 (0.51–13.67) in the propensity score-matched cohort]. In all the other individual cancers examined, no association with ranitidine use was observed either. The higher cumulative exposure to ranitidine, measured by duration in days and dose in milligrams, did not increase the risk of developing cancer than with other H2RAs use (Table 3).

**Table 2:** Risk of all cancers and individual cancers in ranitidine users compared with that in other H2RA users in overall cohort and propensity score matched cohort.

Outcome	Exposure group	Overall cohort					Propensity score matched cohort				
		No. of events	1000 person years	Incidence rate per 1000 person years (95% CI)	Crude HR	Adjusted HR	No. of events	1000 Person years	Incidence rate per 1000 person years (95% CI)	Crude HR	Adjusted HR^†^
All cancer	Ranitidine	244	86.1	2.8 (2.5–3.2)	1.01 (0.89–1.16)	0.95 (0.83–1.09)	205	70.0	2.9 (2.5–3.4)	1.00 (0.83–1.21)	0.98 (0.81–1.20)
	Other H2RAs	1731	529.3	3.3 (3.1–3.4)	Reference	Reference	216	72.9	3.0 (2.6–3.4)	Reference	Reference
Oesophagus	Ranitidine	4	88.2	0.05 (0.01–0.12)	0.80 (0.28–2.27)	0.58 (0.20–1.69)	4	71.8	0.06 (0.02–0.14)	0.83 (0.22–3.09)	0.81 (0.21–3.10)
	Other H2RAs	34	542.3	0.06 (0.04–0.09)	Reference	Reference	5	74.7	0.07 (0.02–0.16)	Reference	Reference
Gastric	Ranitidine	41	87.9	0.47 (0.33–0.63)	1.12 (0.80–1.56)	0.94 (0.67–1.33)	37	71.6	0.52 (0.36–0.71)	1.08 (0.68–1.71)	1.04 (0.65–1.64)
	Other H2RAs	261	540.3	0.48 (0.43–0.55)	Reference	Reference	36	74.4	0.48 (0.34–0.67)	Reference	Reference
Colorectal	Ranitidine	35	88.0	0.40 (0.28–0.55)	0.93 (0.65–1.33)	0.78 (0.54–1.13)	29	71.7	0.40 (0.27–0.58)	0.78 (0.48–1.26)	0.76 (0.47–1.23)
	Other H2RAs	269	540.4	0.50 (0.44–0.56)	Reference	Reference	39	74.5	0.52 (0.37–0.72)	Reference	Reference
Liver	Ranitidine	25	87.9	0.28 (0.18–0.42)	0.94 (0.62–1.43)	0.82 (0.53–1.27)	23	71.6	0.32 (0.20–0.48)	0.93 (0.53–1.62)	0.90 (0.51–1.58)
	Other H2RAs	185	541.1	0.34 (0.29–0.39)	Reference	Reference	26	74.6	0.35 (0.23–0.51)	Reference	Reference
Pancreatic	Ranitidine	4	88.2	0.05 (0.01–0.12)	0.73 (0.26–2.05)	0.69 (0.24–1.99)	4	71.8	0.06 (0.02–0.14)	1.40 (0.31–6.26)	1.32 (0.29–5.94)
	Other H2RAs	40	542.2	0.07 (0.05–0.10)	Reference	Reference	3	74.7	0.04 (0.01–0.12)	Reference	Reference
Lung	Ranitidine	1	88.3	0.01 (0.00–0.06)	0.27 (0.04–1.96)	0.28 (0.04–2.10)	1	71.9	0.01 (0.00–0.08)	0.36 (0.04–3.42)	0.35 (0.04–3.37)
	Other H2RAs	29	542.3	0.05 (0.04–0.08)	Reference	Reference	3	74.7	0.04 (0.01–0.12)	Reference	Reference
Kidney	Ranitidine	7	88.2	0.08 (0.03–0.16)	1.93 (0.83–4.49)	1.49 (0.61–3.61)	5	71.9	0.07 (0.02–0.16)	2.62 (0.51–13.50)	2.65 (0.51–13.67)
	Other H2RAs	25	542.3	0.05 (0.03–0.07)	Reference	Reference	2	74.7	0.03 (0.00–0.10)	Reference	Reference
Bladder	Ranitidine	5	88.2	0.06 (0.02–0.13)	0.88 (0.34–2.24)	0.82 (0.31–2.17)	2	71.9	0.03 (0.00–0.10)	0.53 (0.10–2.90)	0.52 (0.10–2.83)
	Other H2RAs	39	542.2	0.07 (0.05–0.10)	Reference	Reference	4	74.7	0.05 (0.01–0.14)	Reference	Reference
Thyroid	Ranitidine	22	88.1	0.25 (0.16–0.38)	0.84 (0.54–1.31)	1.04 (0.66–1.65)	18	71.8	0.25 (0.15–0.40)	0.76 (0.41–1.39)	0.77 (0.42–1.40)
	Other H2RAs	192	541.3	0.35 (0.31–0.41)	Reference	Reference	25	74.6	0.34 (0.22–0.49)	Reference	Reference

*CI* confidence interval, *H2RA* histamine-2 receptor antagonist, *HR* hazard ratio.

^†^Adjusted for age, sex, type of health insurance, income level, region, year of index entry, COPD, alcohol related disorders, hypertension, diabetes, severe liver disease, obesity.

**Table 3:** Risk of all cancers in ranitidine users compared with that in other H2RA users by cumulative exposure duration and dose in the propensity score matched cohort.

	No. of events	1000 person years	Incidence rate per 1000 person years (95% CI)	Crude HR (95% CI)	Adjusted HR (95% CI)^†^
**Cumulative exposure duration (days)**					
Other H2RAs	216	72.9	3.0 (2.6–3.4)		
**Ranitidine**					
≤ 14 days	93	37.2	2.5 (2.0–3.1)	0.88 (0.69–1.12)	0.94 (0.74–1.20)
15–30 days	44	13.4	3.3 (2.4–4.4)	1.11 (0.80–1.53)	1.11 (0.80–1.53)
31–60 days	34	8.6	4.0 (2.7–5.5)	1.30 (0.91–1.87)	1.24 (0.86–1.78)
61–90 days	10	3.2	3.1 (1.5–5.7)	1.02 (0.54–1.93)	0.85 (0.45–1.60)
91–180 days	14	3.2	4.4 (2.4–7.3)	1.41 (0.82–2.43)	1.18 (0.69–2.03)
> 180 days	10	4.3	2.3 (1.1–4.3)	0.75 (0.40–1.42)	0.53 (0.28–1.01)
**Cumulative dose (defined daily dose, DDD)**					
Other H2RAs	216	72.9	3.0 (2.6–3.4)		
**Ranitidine**					
< 6 DDD	65	25.0	2.6 (2.0–3.3)	0.92 (0.70–1.22)	1.01 (0.77–1.34)
6–50 DDD	109	35.0	3.1 (2.6–3.8)	1.05 (0.84–1.33)	1.05 (0.83–1.32)
> 50 DDD	31	10.0	3.1 (2.1–4.4)	1.00 (0.69–1.46)	0.78 (0.53–1.14)

*CI* confidence interval, *H2RA* histamine-2 receptor antagonist.

^†^Adjusted for age, sex, type of health insurance, income level, region, index year, COPD, alcohol related disorders, hypertension, diabetes, severe liver disease, obesity.

In stratified analysis, ranitidine use was not associated with risk of cancer in any stratum, and indicated no significant interaction. However, relatively high HR with wide confidence interval was estimated in the women [adjusted HR (95% CI), 1.28 (0.92–1.78)] given the statistical insignificance. (Table 4).

**Table 4:** Sub-group-based stratified analysis to examine the risk of all cancers in ranitidine users compared with that in other H2RA users in the propensity score matched cohort.

Variable	No. of patients	No. of event	Incidence rate per 1000 person years	Crude HR (95% CI)	Adjusted HR^†^ (95% CI)	p-value for interaction
**Sex**						0.078
Male	8199	128	2.9	0.88 (0.70–1.12)	0.84 (0.66–1.07)	
Female	4481	77	3.1	1.28 (0.92–1.78)	1.28 (0.92–1.78)	
**Age at index**						0.137
40–64	10 017	112	2.1	1.05 (0.81–1.37)	1.06 (0.82–1.38)	
65–84	2142	80	6.5	0.92 (0.68–1.24)	0.89 (0.66–1.20)	
85 +	521	13	3.3	1.01 (0.47–2.18)	0.88 (0.40–1.97)	
**Type of health insurance**						0.600
Health insurance	12 155	194	2.9	0.99 (0.81–1.20)	0.97 (0.80–1.18)	
Medical aid	525	11	3.2	1.21 (0.51–2.85)	1.30 (0.55–3.09)	
**Income level**						0.470
1st quartile (most deprived)	1992	32	2.9	0.84 (0.53–1.34)	0.82 (0.52–1.31)	
2nd quartile	3333	53	3.0	1.13 (0.77–1.66)	1.11 (0.75–1.64)	
3rd quartile	4472	65	2.6	1.18 (0.83–1.68)	1.15 (0.81–1.64)	
4th quartile (most affluent)	2883	55	3.3	0.85 (0.59–1.21)	0.83 (0.58–1.19)	
**Region**						0.798
Capital area	5504	78	2.6	0.98 (0.72–1.33)	0.95 (0.70–1.30)	
Other regions	7176	127	3.2	1.01 (0.79–1.29)	1.00 (0.78–1.27)	
**Comorbidities**						
Hypertension						0.093
No	873	24	4.3	1.05 (0.86–1.30)	1.05 (0.86–1.29)	
Yes	11 807	181	2.8	0.64 (0.38–1.09)	0.60 (0.35–1.02)	
Diabetes						0.103
No	431	13	4.6	1.04 (0.85–1.27)	1.04 (0.85–1.26)	
Yes	12 249	192	2.9	0.54 (0.27–1.09)	0.52 (0.26–1.07)	
Severe liver disease						0.647
No	172	8	6.7	0.99 (0.82–1.20)	0.98 (0.80–1.19)	
Yes	12 508	197	2.9	1.14 (0.40–3.30)	2.12 (0.61–7.37)	
Chronic pulmonary disease						0.639
No	392	11	4.2 (2.1–7.6)	0.89 (0.39–2.03)	0.78 (0.47–2.70)	
Yes	12 288	194	2.9 (2.5–3.3)	1.01 (0.83–1.23)	1.00 (0.82–1.21)	
**Comedications**						
Glucocorticoids for systemic use						0.738
Yes	1159	19	2.7 (1.6–4.2)	0.96 (0.52–1.79)	0.78 (0.41–1.47)	
No	11 521	186	3.0 (2.5–3.4)	1.01 (0.82–1.23)	1.00 (0.82–1.22)	
Non-aspirin NSAID						0.009
Yes	3009	77	4.3 (3.4–5.3)	1.48 (1.05–2.09)	1.48 (1.05–2.10)	
No	9671	128	2.5 (2.1–2.9)	0.82 (0.66–1.06)	0.82 (0.65–1.04)	

*CI* confidence interval, *H2RA* histamine-2 receptor antagonist, *HR* hazard ratio, *NSAID* non-steroidal anti-inflammatory drugs.

^†^Adjusted for age, sex, type of health insurance, income level, region, index year, COPD, alcohol-related disorders, hypertension, diabetes, severe liver disease, obesity.

The results from the sensitivity analyses I, in which the lag time varied from no lag-time to six years, did not differ from the main findings. This finding suggested that no association existed between the use of ranitidine and cancer risk at any lag-time setting (Table 5). However, despite the lack of statistical significance, a slightly higher risk of cancer was present in the six-year lag-time setting in the propensity score-matched analysis [HR (95% CI), 1.12 (0.79–1.59)] (Table 5). Sensitivity analysis II, which included all subjects who experienced switching, and sensitivity analysis III, which included all patients with cancer during lag-time, provided similar results to those of the primary analysis (Tables 6, 7). The results of the sensitivity study IV, which excluded those who had only ever received one prescription, and V, which extended the look-back time for comorbidities and co-medications to two years, did not differ from the primary results (Supplementary Tables 2 and 3). Supplementary analysis did not show cancer risk attributed to ranitidine use (Supplementary Table 2).

**Table 5:** The sensitivity analysis I: association between ranitidine and cancer risk by varying lag-time in propensity score matched cohort.

Lag-time	No. of events	1000 person years	Incidence rate per 1000 person years (95% CI)	Crude HR	Adjusted HR^†^
**No lag-time applied**					
Ranitidine	415	70.0	5.9 (5.4–6.5)	1.09 (0.95–1.25)	1.08 (0.94–1.24)
Other H2RAs	392	72.9	5.4 (4.9–5.9)	Reference	Reference
**Two years**					
Ranitidine	205	70.0	2.9 (2.5–3.4)	1.00 (0.83–1.21)	0.98 (0.81–1.20)
Other H2RAs	216	72.9	3.0 (2.6–3.4)	Reference	Reference
**Four years**					
Ranitidine	112	70.0	1.6 (1.3–1.9)	1.03 (0.79–1.33)	1.02 (0.79–1.33)
Other H2RAs	117	72.9	1.6 (1.3–1.9)	Reference	Reference
**Six years**					
Ranitidine	63	70.0	0.9 (0.7–1.2)	1.12 (0.79–1.59)	1.12 (0.79–1.59)
Other H2RAs	61	72.9	0.8 (0.6–1.1)	Reference	Reference

*CI* confidence interval, *H2RA* histamine-2 receptor antagonist.

^†^Adjusted for age, sex, type of health insurance, income level, region, index year, COPD, alcohol-related disorders, hypertension, diabetes, severe liver disease, obesity.

**Table 6:** Sensitivity analysis II: risk of all cancers in ranitidine users compared with that in other H2RA users, including subjects who have switched between ranitidine and other H2RAs, in overall cohort and propensity matched cohort.

Exposure group	No. of patients	No. of events	1000 Person years	Incidence rate per 1000 person years (95% CI)	Crude HR	Adjusted HR^†^
**Overall cohort**						
Ranitidine	61 560	2286	441	5.2 (5.0–5.4)	1.07 (1.04–1.12)	1.00 (0.96–1.05)
Other H2RAs	227 114	10,217	1970	5.2 (5.1–5.3)	Reference	Reference
**Propensity score matched cohort**						
Ranitidine	56 507	2208	422	5.2 (5.0–5.5)	0.99 (0.93–1.05)	0.98 (0.92–1.03)
Other H2RAs	56 507	2275	429	5.3 (5.1–5.5)	Reference	Reference

*CI* confidence interval, *H2RA* histamine-2 receptor antagonist.

^†^Adjusted for age, sex, type of health insurance, income level, region, index year, COPD, alcohol-related disorders, hypertension, diabetes, severe liver disease, obesity.

**Table 7:** Sensitivity analysis III: Association between ranitidine use and cancer risk in which the person-time of patients diagnosed with cancer during the two-year lag-time period was excluded from the person-time summation.

Exposure group	No. of patients	No. of events	1000 person years	Incidence rate per 1000 person years (95% CI)	Crude HR	Adjusted HR^†^
**Overall cohort**						
Ranitidine	18 373	244	85.9	2.84 (2.50–3.22)	1.01 (0.89–1.16)	0.95 (0.83–1.09)
Other H2RAs	68 407	1731	528.6	3.27 (3.12–3.43)	Reference	Reference
**Propensity score matched cohort**						
Ranitidine	12 470	205	69.9	2.93 (2.55–3.36)	1.00 (0.83–1.21)	0.98 (0.81–1.19)
Other H2RAs	12 504	216	72.8	2.97 (2.58–3.39)	Reference	Reference

*CI* confidence interval, *H2RA* histamine-2 receptor antagonist.

^†^Adjusted for age, sex, type of health insurance, income level, region, index year, COPD, alcohol-related disorders, hypertension, diabetes, severe liver disease, obesity.

## Discussion

As unacceptable levels of NDMA impurities were detected in many ranitidine products in 2019, health authorities, such as the FDA, EMA, and MFDS, took measures to withdraw ranitidine products from the market. In this population-based study, the association between the use of ranitidine and cancer risk was investigated. This study provided no evidence of association of NDMA impurities in ranitidine products with the risk of cancer. Little can be suggested about individual cancers due to the lack of power, these results remained consistent in the stratified analysis and several sensitivity analyses. The cohort’s follow-up was 5.5 years, not long enough to assess long-term cancer risk. The findings should be considered as short-term cancer risk.

### Comparison with other studies

NDMA is a probable human carcinogen, based on laboratory studies, so its effect on humans rely on observational studies. Most studies in humans are nutritional epidemiological research, focusing on the dietary consumption of NDMA^36–42^, while some studies have evaluated the impacts of occupational exposure^42,43^. There are varied results depending on the study design, exposure level, and carcinoma of interest, but significant results have been suggested in a large number of studies.

According to a recent meta-analysis that evaluated the relationship between NDMA and gastric cancer by integrating 11 studies, NDMA increased the risk of gastric cancer [HR (95% CI), 1.34 (1.02 to 1.76)]^44^. Other studies on dietary intake of NDMA have shown significant findings in lung cancer^40^, colorectal cancer^40^, pancreatic cancer^45^, and upper aerodigestive tract cancer (laryngeal, esophageal, and oral)^38^. In a recent study with a long follow-up period (49 years) and lag-time (15 years) to evaluate the exposure–response relationship between occupational exposures to N-nitrosamines and cancer mortality in the UK rubber industry, NDMA exposure was associated with overall cancers [HR (95% CI), 2.08 (1.96–2.21)] and cancers of the bladder, stomach, leukemia, multiple myeloma, prostate, and liver^43^. Another analysis involving a 10-year lag time cohort of rubber employees found that exposure to high concentrations of nitrosamine, calculated as NDMA and N-nitrosomorpholine, was correlated with an increased mortality from oesophagus, oral cavity, and pharynx cancers^42^.

Our findings were distinct from those of the previous nutritional or occupational epidemiological studies stated above; however, a direct comparison with our study would not be suitable. Confounding is a specific challenge in nutritional epidemiological research because dietary components are correlated, making it difficult to distinguish their impacts. In addition, the self-administered tools for dietary NDMA exposure measurements, such as food frequency questionnaires, have a high risk of recall bias^46^. The association between dietary NDMA consumption and gastric cancer risk was primarily noted in case–control studies rather than in cohort studies^44^, indicating the likelihood of differential misclassification in exposure due to recall bias. Regarding studies on occupational NDMA exposure and cancer risk, exposure measures based on airborne concentrations and plant-specific conditions are far from the line of our investigation.

The issue of NDMA contaminated ranitidine was very recently, in 2019. To our knowledge, a few studies have investigated the link between the potential NDMA impurities in ranitidine and overall cancer risk^16–18^. The estimates of overall cancer risk levels in our study were very close to those reported in these studies. Two previous studies are similar to our study in that they used claim data. The Japanese study has a short follow-up period, which can provide insight on short-term cancer risk, and has limitations in terms of external validity as it only targets employed workers and their families^16^. The study in Korea secured comparability with famotidine users as a control group. However, the researchers noted that simply matching by gender, age, cumulative time, and diabetes mellitus provided insufficient control for potential confounding variables^17^. In the study using UK Biobank, exposure was self-reported, with little information^18^. In the case of individual cancers, we did not found statistical significance in any single carcinoma tested which is in line with the previous studies. However, the studies had the same limitation (power was not secured due to the small number of subjects and events). Notably, a recent nested case–control study showed the link between the use of ranitidine and the risk of bladder cancer^23^.

In the duration/dose response analysis, the group with the longest use period (> 180 days) or highest cumulative dose (> 50 DDD) showed a lower HR (HR [95% CI], 0.53 [0.28–1.01], 0.78 [0.53–1.14], respectively). These results are similar to those of a previous ranitidine/nizatidine study showing the lowest HR in the highest cumulative usage group (above 730 defined daily dose) [HR (95% CI), 0.83 (0.45–1.55)], although it did not reach statistical significance^16^. Termination of exposure may be related to the presence of disease (a variant of the ‘healthy worker effect’)^47^. Rather than interpreting these results as a possible reverse causation, it is likely that people with large amounts of cumulative ranitidine use may be due to an increase in person-time because cancer has not yet occurred. This is likely to be less biased if it was possible to determine the cumulative exposure to ranitidine with sufficient exposure window before follow-up, and to follow the cohort long after termination of exposure.

In this study, kidney cancer presented the highest HR among the examined cancer sites [HR (95% CI), 2.65 (0.51–13.67) in the propensity score-matched cohort], although sufficient power was not ensured. A laboratory study that analyzed NDMA levels after oral intake of ranitidine reported that urinary excretion of 150 mg ranitidine after 24 h increased by 400 times, from 110 to 47,600 ng^48^. Research on the relationship between ranitidine use and kidney cancer needs to be conducted more closely in the future.

The FDA has stated that the levels of NDMA in ranitidine are close to those of common foods items such as grilled or smoked meat^49^. The FDA determined that ingestion of ≤ 96 ng or 0.32 ppm of NDMA per day should be reasonably safe in humans^49^. FDA has set the same acceptable daily intake limit for NDMA for ranitidine^49^. However, the exposure to NDMA from taking ranitidine is likely to be very high in some patients. For instance, if a patient had taken a ranitidine product containing 14.68 ppm of NDMA, which is the minimum detected amount of a specific company's raw ranitidine substance sample in Korea^15^, for 1 year consecutively, that is the same as taking a quantity of 46 times the FDA's acceptable limit (0.32 ppm) throughout the year. Considering that clinicians usually keep prescribing a specific pharmaceutical brand in Korea, this level of exposure will not be very rare. For comparison, in nutritional epidemiologic studies that explored the risk of dietary intake of NDMA, the cancer risk in the highest tertile or quintile was 1.4^37^, 1.96^50^, or 2.43^51^ compared to that in the lowest exposure group. However, the daily dietary exposure to NDMA estimated in these studies ranged from 190 to 240 ng/day, which is only 2–4 times the acceptable limit of FDA.

### Strengths and limitations

This study has several strengths. First, through propensity score analysis and employing active control, comparability was enhanced, and confounding by indication could be lessened. Second, misclassification by switching was prevented by excluding patients who had experienced switching between ranitidine and active comparator. Third, as the risk window and latency were difficult to determine, the latency period was included by placing multiple risk windows for a lag time of up to 6 years. Finally, the possible misclassification of outcome variables was lowered by utilizing the V code when identifying cancer occurrence. In a study using Korean National Health Insurance (NHI) claims database, cases registered as pancreatic cancer by ICD-10 and V codes without pathologic confirmation achieved a high accuracy including a positive predictive value of 98.08%. Whether ranitidine, which has been used by many people for a long time, raise the risk of cancer is a critical question that requires long-term investigation. We expect that our study reduce uncertainty by confirming the findings of a limited existing studies that suggested no link between ranitidine use and short term cancer risk. Nonetheless, the results of this study should be interpreted with caution due to certain significant limitations. First, the follow-up period was not long enough to confirm the relationship between NDMA and cancer incidence, which is a critical disadvantage of our study. In addition, NDMA acts as an initiator and takes longer from exposure to cancer, which contrasts with the fact that drug exposure mostly serves as a promotor when exerting cancer development^52^. Among the prior nutritional epidemiologic studies, the follow-up periods in cohort studies suggesting significant association ranged from 11.4 to 24 years^41,50,51,53^. Due to the variations in research methodologies, direct comparisons may not be feasible. However, in the two studies with the most extended follow-up periods (18 years and 24 years), the HR for cancer incidence was 2.0 (gastric cancer) and 2.12 (colorectal cancer), respectively^41,50^, which are higher than those reported in other studies. On the other hand, a research with 6.6 years of follow-up did not indicate relevance^54^. Second, the risk of individual cancers, high-dose users, and different subgroups was explored in our research. However, the results generally failed to secure statistical power and did not yield sufficiently valuable evidence. The substantial loss of eligible subjects can be attributed to implementing an active comparator and excluding those who experienced switching. Third, the level of NDMA impurities in ranitidine varies by product, defined as exposure in this study, may not accurately represent NDMA exposure. Fourth, the chances of residual confounding may remain because the potential confounding factors, such as food, cigarette smoking, and alcohol consumption, were not included. Fifth, since prescription data from the hospitals’ claim data was used in this study, we could not verify if the prescriptions were actually filled. Finally, one year look-back period for comorbidities and co-medication was insufficient. Finally, measuring the cumulative use from the follow-up start date may lead to immortal time bias.

In summary, no association was found between ranitidine with potential NDMA impurities and the risk of overall cancer and major individual malignancies. Our study supported the findings of other investigations after rigorous controlling for confounding variables to ensure comparability in the population where ranitidine use was highly prevalent. The findings should be interpreted with caution considering insufficient follow-up, and longer follow-up are required to estimate long-term risk of cancer.

## Supplementary Information

Supplementary Tables.

## Data Availability

The health insurance claims database of the National Health Insurance Service can be accessed at https://nhiss.nhis.or.kr/bd/ab/bdaba022eng.do.
