# Supplementary material for: Association between ranitidine use with potential NDMA impurities and risk of cancer in Korea
Source: Sci Rep. 2022 Dec 27;12:22396. doi: 10.1038/s41598-022-26691-0 (PMC9794704; doi:10.1038/s41598-022-26691-0)
Supplement: Supplementary file 1 — Supplementary Tables. [file 41598_2022_26691_MOESM1_ESM.docx]

Association Between Ranitidine Use with Potential NDMA Impurities and Risk of Cancer in Korea

**Supplementary tables**

Supplementary table 1. ICD-10 codes of co-morbidities as potential confounders

| **Co-morbidity** | **ICD-10 codes** |
| --- | --- |
| Hypertension | I10-I15 |
| Ulcerative colitis | K50-K51 |
| Obesity | E65-E68 |
| Chronic/Severe liver disease | K70-K74 |
| Diabetes type I and II | E10-E14 |
| Chronic obstructive pulmonary disease | J42–J44 J40-J47 |
| Kidney diseases | N00-N19 |
| Disorders of gallbladder  biliary tract and pancreas | K80-K87 |
| Congestive heart failure | I11.0, I13.0, I13.2, I42, I43, I50, I51.7 |
| Ischemic heart disease | I20-I25 |
| Atrial fibrillation | I48 |
| Stroke | I64 |
| Alcohol related disease | E244, E529A, G31.2, G62.1, G72.1, I42.6, F10.2, K29.2, K70, K86.0, T519, Z502, Z714, Z72.1 |

Abbreviations: ICD-10, international classification of disease 10^th^ revision

Supplementary table 2. Sensitivity analysis Ⅳ: Association between ranitidine use and cancer risk in which patients who received prescription of ranitidine or active comparator once were excluded to limit inclusion of non-compliant users

|  | Exposure group | No. of patients | No. of events | 1000 person years | Incidence rate per 1000 person years (95% CI) | Crude HR | Adjusted HR ^†^ |
| --- | --- | --- | --- | --- | --- | --- | --- |
| Overall cohort | | |  |  |  |  |  |
|  | Ranitidine | 8 338 | 171 | 49.4 | 3.46 (2.96 to 4.02) | 1.05 (0.89-1.25) | 1.05 (0.89 to 1.26) |
|  | Other H2RAs | 23 888 | 487 | 146.6 | 3.32 (3.03 to 3.63) | Reference | Reference |
| Propensity score matched cohort | | |  |  |  |  |  |
|  | Ranitidine | 8 338 | 171 | 49.4 | 3.46 (2.96 to 4.02) | 1.05 (0.85-1.30) | 1.05 (0.85-1.30) |
|  | Other H2RAs | 8 338 | 170 | 51.2 | 3.32 (2.84 to 3.86) | Reference | Reference |

Abbreviations: CI, confidence interval; H2RA, histamine-2 receptor antagonist

^†^ Adjusted for age, sex, type of health insurance, income level, region, index year, COPD, alcohol-related disorders, hypertension, diabetes, severe liver disease, obesity

Supplementary table 3. Sensitivity analysis Ⅴ: Association between ranitidine use and cancer risk in which the look-back period for comorbidities and co-medications was extended to two years

|  | Exposure group | No. of patients | No. of events | 1000 person years | Incidence rate per 1000 person years (95% CI) | Crude HR | Adjusted HR |
| --- | --- | --- | --- | --- | --- | --- | --- |
| Overall cohort | | |  |  |  |  |  |
|  | Ranitidine | 18 666 | 244 | 85.9 | 2.84 (2.50 to 3.22) | 1.01 (0.89-1.16) | 0.95 (0.83 to 1.09) |
|  | Other H2RAs | 69 294 | 1731 | 528.6 | 3.27 (3.12 to 3.43) | Reference | Reference |
| Propensity score matched cohort | | |  |  |  |  |  |
|  | Ranitidine | 12 758 | 216 | 71.7 | 3.01 (2.62 to 3.44) | 1.03 (0.86 to 1.25) | 1.02 (0.84 to 1.23) |
|  | Other H2RAs | 12 758 | 215 | 73.3 | 2.93 (2.55 to 3.35) | Reference | Reference |

Abbreviations: CI, confidence interval; H2RA, histamine-2 receptor antagonist

^‡^ Adjusted for age, sex, type of health insurance, income level, region, index year, COPD, alcohol-related disorders, hypertension, diabetes, severe liver disease, obesity

Supplementary table 4. Attributable risk and population attributable risk for all cancers and individual cancers in ranitidine users compared with other H2RA users, in overall cohort and propensity score matched cohort.

|  |  | **Overall cohort** | | | |  | **PS matching cohort** | | | |
| --- | --- | --- | --- | --- | --- | --- | --- | --- | --- | --- |
| **Outcome** | **Exposure** | **No. of event** | **Incidence rate** | **AR (%)** ^†^ | **PAR (%)** ^‡^ |  | **No. of event** | **Incidence rate** | **AR (%)** ^†^ | **PAR (%)** ^‡^ |
| Overall cancer | Ranitidine | 244 | 2.83 | -15.55 | -1.90 |  | 205 | 2.93 | -16.67 | -4.21 |
|  | Other H2RAs | 1,731 | 3.27 |  |  |  | 216 | 2.96 |  |  |
| Esophagus | Ranitidine | 4 | 0.05 | -20.00 | -4.03 |  | 4 | 0.06 | -16.67 | -8.95 |
|  | Other H2RAs | 34 | 0.06 |  |  |  | 5 | 0.07 |  |  |
| Gastric | Ranitidine | 41 | 0.47 | -2.13 | -0.48 |  | 37 | 0.52 | 7.69 | 3.23 |
|  | Other H2RAs | 261 | 0.48 |  |  |  | 36 | 0.48 |  |  |
| Colorectal | Ranitidine | 35 | 0.40 | -25.00 | -2.90 |  | 29 | 0.40 | -30.00 | -12.55 |
|  | Other H2RAs | 269 | 0.50 |  |  |  | 39 | 0.52 |  |  |
| Liver | Ranitidine | 25 | 0.28 | -21.43 | -2.41 |  | 23 | 0.32 | -9.37 | -3.99 |
|  | Other H2RAs | 185 | 0.34 |  |  |  | 26 | 0.35 |  |  |
| Pancreatic | Ranitidine | 4 | 0.05 | -40.00 | -5.70 |  | 4 | 0.06 | 33.33 | 15.95 |
|  | Other H2RAs | 40 | 0.07 |  |  |  | 3 | 0.04 |  |  |
| Lung | Ranitidine | 1 | 0.01 | -400.00 | -12.41 |  | 1 | 0.01 | -300.00 | -47.19 |
|  | Other H2RAs | 29 | 0.05 |  |  |  | 3 | 0.04 |  |  |
| Kidney | Ranitidine | 7 | 0.08 | 37.50 | 9.17 |  | 5 | 0.07 | 57.14 | 43.93 |
|  | Other H2RAs | 25 | 0.05 |  |  |  | 2 | 0.03 |  |  |
| Bladder | Ranitidine | 5 | 0.06 | -16.67 | -3.05 |  | 2 | 0.03 | -66.67 | -30.83 |
|  | Other H2RAs | 39 | 0.07 |  |  |  | 4 | 0.05 |  |  |
| Thyroid | Ranitidine | 22 | 0.25 | -40.00 | -4.32 |  | 18 | 0.25 | -36.00 | -14.10 |
|  | Other H2RAs | 192 | 0.35 |  |  |  | 25 | 0.34 |  |  |

Abbreviations: AR, attributable risk; PAR, population attributable risk

^†^ AR (%) = (Incidence _exposed_ – Incidence _nonexposed_) /Incidence _exposed_

^‡^ PAR (%) = (Incidence _population_ – Incidence _unexposed_)/Incidence _population_

Proportion of ranitidine users used for calculating Incidence _population_ was 13.6%, which was referenced from the Ministry of Food and Drug Safety <https://www.mfds.go.kr/docviewer/skin/doc.html?fn=20190926064954710.hwp&rs=/docviewer/result/ntc0021/43717/1/201909> and considering non-prescription ranitidine use. The incidence in the total population (Incidence _population_) was calculated as follows: $\left( incidence in ranitidine exposed group \right)\left( \% ranitidine users in population \right)$+$\left( incidence in Other H2RA exposed group \right)\left( \% ranitidine non-users in population \right)$
